# Supplementary material for: Accurate and versatile 3D segmentation of plant tissues at cellular resolution
Source: eLife. 2020 Jul 29;9:e57613. doi: 10.7554/eLife.57613 (PMC7447435; doi:10.7554/eLife.57613)

PlantSeg - Analysis of daughter cells volume ratio after first assymetric division of lateral root founder cells (Figure 7)

This document presents the analysis of daughter cells volume ratio after first assymetric division of founder cells as in Figure 7C.

Library used

```
library(tidyverse)
```

Load volume data

First we load data containing for each of the three movies segmented by `PlantSeg`: the volume of each cells.

```
movie1_vol <- read.csv("movie1_bioutifoul_cell_volumes.csv")
movie2_vol <- read.csv("movie2_marvelous_cell_volumes.csv")
movie3_vol <- read.csv("movie3_nice_cell_volumes.csv")
```

We then aggregate all three in a single dataframe:

```
all_vol <- bind_rows(movie1_vol, movie2_vol, movie3_vol)
```

Each cell is identified by a *label*, unique to each movie. Each entry also contains a unique *ID* obtained by concatenation of the movie nickname, frame of division and label of the dividing cell.

Identifiying the founder cells undergoing their first assymetric division

Human expert screened each segmented movie and compiled a list of all founder cells undergoing their first assymetric division and their daugther cells, recording in particular their *label*. Example of such divisions is shown on Figure 7B.

```
acd_list <- read.csv2("ACD_list.csv")
```

We create now a unique *ID* for each entry by concatenation of the movie nickname, frame of division and label of the dividing cell.

```
acd_list <- acd_list %>% mutate(ID=paste0(MovieName,"_frame_",Frame,"_label_",Label))
```

We now retrieve from `all_vol` table the volume of the daughter cells present in `acd_list`, using the *ID* as key:

```
acd_list_volumes <- acd_list %>% inner_join(all_vol, acd_list, by="ID")
```

Compute and visualise ratio of volume between the two daughter cells of an asymmetric cell division

To compute the ratio of volume between the two daughter cells of an asymmetric cell division, we group daughter cells together (same `ParentID.x`), sort them by volume (descending) and compute the ratio between the two volume, finally we re-arrange the cells chronologically and by common parent.

```
acd_list_volumes_ratio <- acd_list_volumes %>% group_by(ParentID.x) %>% arrange (-Volume) %>%
  mutate(Ratio=Volume/lag(Volume)) %>% arrange(MovieName.y, Frame.x, ParentID.x)
```

Finally we visualise the distribution of ratio using a half-violin/half dot plot geometry.

```
source('geom_flat_violin.R')
ggplot(data = acd_list_volumes_ratio %>% filter(!is.na(Ratio)),  mapping = aes(x = MovieName.x, y = Rat
  geom_flat_violin(scale = "count", trim = FALSE, fill = "grey") +
  stat_summary(fun.data = mean_sdl, fun.args = list(mult = 1), geom = "pointrange", position = position
  geom_dotplot(binaxis = "y", dotsize = 2, stackdir = "down", binwidth = 0.02, position = position_nudg
  theme_light()+
  theme(legend.position = "none") +
  labs(x="", y = "Daughter cells volume ratio (a.u.)")+
  scale_x_discrete(labels = c("#1", "#2", "#3"))
```

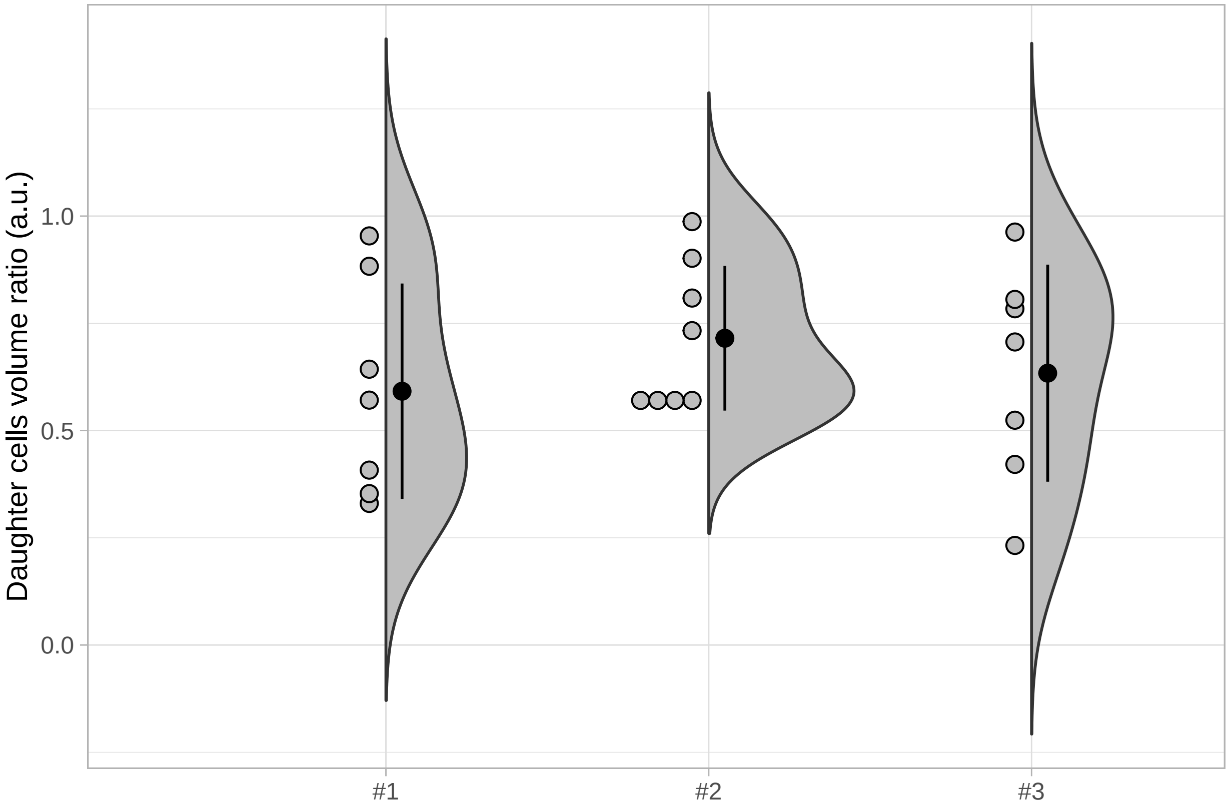

Supplement: Figure 7—source data 1. — A detailed description of how to generate the pane C can be found in 'Figure 7C.pdf'. [file elife-57613-fig7-data1.zip › fig7_root/Figure7C.pdf]
