# Supplementary material for: Accurate and versatile 3D segmentation of plant tissues at cellular resolution
Source: eLife. 2020 Jul 29;9:e57613. doi: 10.7554/eLife.57613 (PMC7447435; doi:10.7554/eLife.57613)
Supplement: Figure 9—source data 1. — The archive contains: 'final_mesh_evaluation - Sheet1.csv' - CSV file with evaluation scores computed on individual meshes, 'Mesh_boxplot.pdf' - detailed steps to reproduce the graphs, 'Mesh_boxplot.ipynb' - python script for generating the graph. [file elife-57613-fig9-data1.zip › figure9/Mesh_boxplot.pdf]

Mesh_boxplot

August 13, 2020

[1]:
```
import matplotlib.pyplot as plt
import h5py
import numpy as np
import csv
from collections import defaultdict
import matplotlib.patches as mpatches
figure_number = "figure_meshes"
```

[2]:
```
rand = defaultdict(list)
in_file = "/home/lcerrone/Downloads/final_mesh_evaluation - Sheet1.csv"

with open(in_file, 'r') as f:
    reader = csv.reader(f, delimiter=',')

    for i, row in enumerate(reader):
        if i!= 0:
            if row[2] in rand:
                rand[row[2]].append(float(row[5]))
            else:
                rand[row[2]] = [float(row[5])]

accuracy = defaultdict(list)
in_file = "/home/lcerrone/Downloads/final_mesh_evaluation - Sheet1.csv"

with open(in_file, 'r') as f:
    reader = csv.reader(f, delimiter=',')

    for i, row in enumerate(reader):
        if i!= 0:
            if row[2] in rand:
                accuracy[row[2]].append(float(row[8]))
            else:
                accuracy[row[2]] = [float(row[8])]
```

[3]:
```
print(rand.keys())
```

```
dict_keys(['pred_*.ply', 'proj*.ply', 'autoSegm.ply'])
```

[4]:
```
rand_list = []
for value in rand.values():
    rand_list.append(value)

accuracy_list = []
for value in accuracy.values():
    accuracy_list.append(value)

print(rand_list)
```

```
[[0.3869969716, 0.2690918342, 0.07944851456, 0.8087619696, 0.08689412524,
0.05171162343], [0.1590771662, 0.1714933677, 0.06546473536, 0.1811655125,
0.05115239322, 0.03741538176], [0.4808004271, 0.2902733082, 0.3605940741,
0.2954310431, 0.4287037157, 0.2668988922]]
```

[5]:
```
fig, ax = plt.subplots(figsize=(18, 12))
#ax.set(xlabel='Specimen',
#       ylabel='Volume $\mathregular{(\mu m^3)}$')

bplot = ax.boxplot(rand_list,
                   notch=False, patch_artist=True, showfliers=True)

bs = 70
for i, value in enumerate(rand_list):
    for j, _v, in enumerate(value):
        sc = ax.scatter(i + 1, _v, c="k",zorder=10, s=bs, marker='o')

#for i, d in enumerate(data):
#    sc = ax.scatter(np.ones(len(d))*(i + 1), d, c="k", zorder=10, s= 5,␣
↪marker="o")

## change outline color, fill color and linewidth of the boxesshowfliers
#for box in bplot["fliers"]:
#    box.set(markeredgecolor="#3bc954", linewidth'#c93b76'=2)

colors = ['#3bc954', '#3bc954', '#3bc954', '#c93b76', '#c93b76', '#c93b76']
for patch, color in zip(bplot['boxes'], colors):
     # change outline color
    patch.set(color='k', linewidth=1.5)
    # change fill color
    patch.set(facecolor = color, alpha=0.8)

## change color and linewidth of the whiskers
colors = ['#3bc954', '#3bc954', '#3bc954',
         '#3bc954', '#3bc954', '#3bc954',
         '#c93b76', '#c93b76', '#c93b76'
         '#c93b76', '#c93b76', '#c93b76']
```

```
for whisker, color in zip(bplot['whiskers'], colors):
    whisker.set(color='k', linewidth=1.5)

## change color and linewidth of the caps
for cap, color in zip(bplot['caps'], colors):
    cap.set(color='k', linewidth=1.5)

## change color and linewidth of the medians
for median in bplot['medians']:
    median.set(color='k', linewidth=1.5)

## change the style of fliers and their fill
for flier in bplot['fliers']:
    flier.set(marker='o', alpha=1, markersize=0)
    flier.set(markerfacecolor='k')

fs2 = 26

ax.yaxis.grid(True)
ax.tick_params(axis='x', labelsize=fs2)
ax.tick_params(axis='y', labelsize=fs2)

plt.xticks([1, 2, 3], ['PredAutoSeg', 'Proj3D', 'RawAutoSeg'])
plt.ylabel("ARand", size=fs2)

green_patch = mpatches.Patch(facecolor="#3bc954", edgecolor='k', linewidth =1.
↪5, label='wt')
purple_patch = mpatches.Patch(facecolor='#c93b76', edgecolor='k', linewidth =1.
↪5, label='mut')
#plt.legend(loc=0, handles=[green_patch, purple_patch], prop={'size': fs2})


fig.savefig(figure_number + "/arand.pdf", bbox_inches='tight')
```

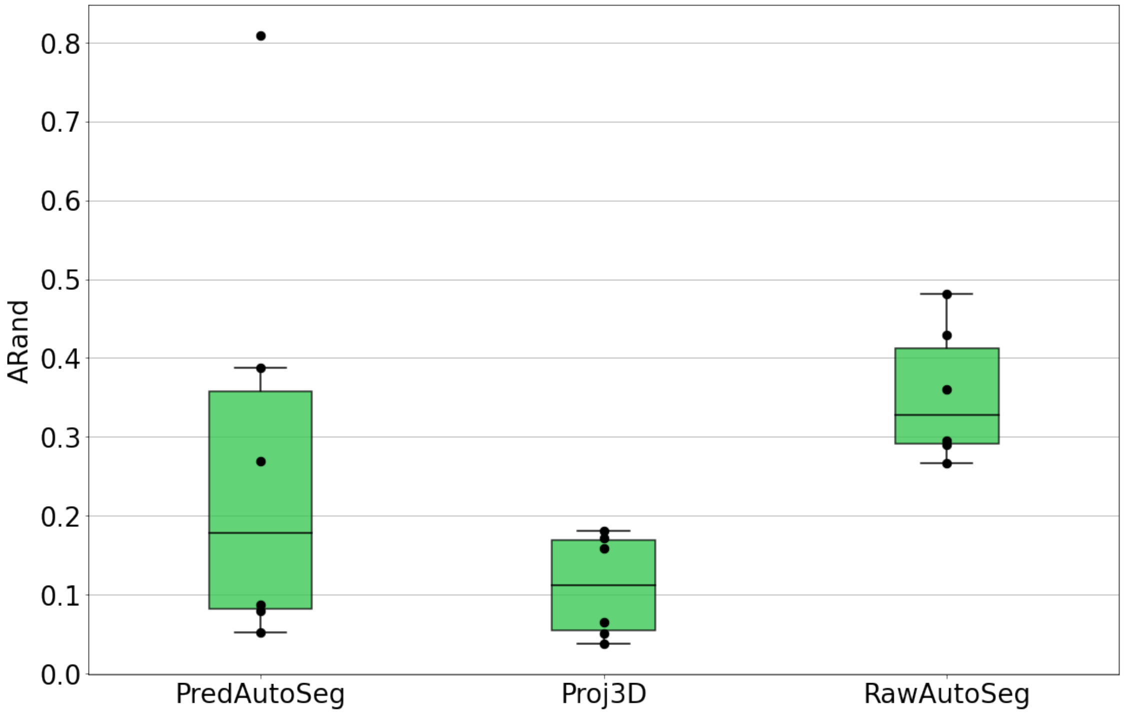

[6]:
```
fig, ax = plt.subplots(figsize=(18, 12))
#ax.set(xlabel='Specimen',
#       ylabel='Volume $\mathregular{(\mu m^3)}$')

bplot = ax.boxplot(accuracy_list,
                   notch=False, patch_artist=True, showfliers=True)

bs = 70
for i, value in enumerate(accuracy_list):
    for j, _v, in enumerate(value):
        sc = ax.scatter(i + 1, _v, c="k",zorder=10, s=bs, marker='o')

#for i, d in enumerate(data):
#    sc = ax.scatter(np.ones(len(d))*(i + 1), d, c="k", zorder=10, s= 5,␣
 ↪marker="o")

## change outline color, fill color and linewidth of the boxesshowfliers
#for box in bplot["fliers"]:
#    box.set(markeredgecolor="#3bc954", linewidth'#c93b76'=2)

colors = ['#3bc954', '#3bc954', '#3bc954', '#c93b76', '#c93b76', '#c93b76']
for patch, color in zip(bplot['boxes'], colors):
     # change outline color
    patch.set(color='k', linewidth=1.5)
```

```
    # change fill color
    patch.set(facecolor = color, alpha=0.8)

## change color and linewidth of the whiskers
colors = ['#3bc954', '#3bc954', '#3bc954',
          '#3bc954', '#3bc954', '#3bc954',
          '#c93b76', '#c93b76', '#c93b76'
          '#c93b76', '#c93b76', '#c93b76']
for whisker, color in zip(bplot['whiskers'], colors):
    whisker.set(color='k', linewidth=1.5)

## change color and linewidth of the caps
for cap, color in zip(bplot['caps'], colors):
    cap.set(color='k', linewidth=1.5)

## change color and linewidth of the medians
for median in bplot['medians']:
    median.set(color='k', linewidth=1.5)

## change the style of fliers and their fill
for flier in bplot['fliers']:
    flier.set(marker='o', alpha=1, markersize=0)
    flier.set(markerfacecolor='k')

fs2 = 26

ax.yaxis.grid(True)
ax.tick_params(axis='x', labelsize=fs2)
ax.tick_params(axis='y', labelsize=fs2)

plt.xticks([1, 2, 3], ['PredAutoSeg', 'Proj3D', 'RawAutoSeg'])
plt.ylabel("Accuracy (%)", size=fs2)

green_patch = mpatches.Patch(facecolor="#3bc954", edgecolor='k', linewidth =1.
 ↪5, label='wt')
purple_patch = mpatches.Patch(facecolor='#c93b76', edgecolor='k', linewidth =1.
 ↪5, label='mut')
#plt.legend(loc=0, handles=[green_patch, purple_patch], prop={'size': fs2})



fig.savefig(figure_number + "/accuracy.pdf", bbox_inches='tight')
```

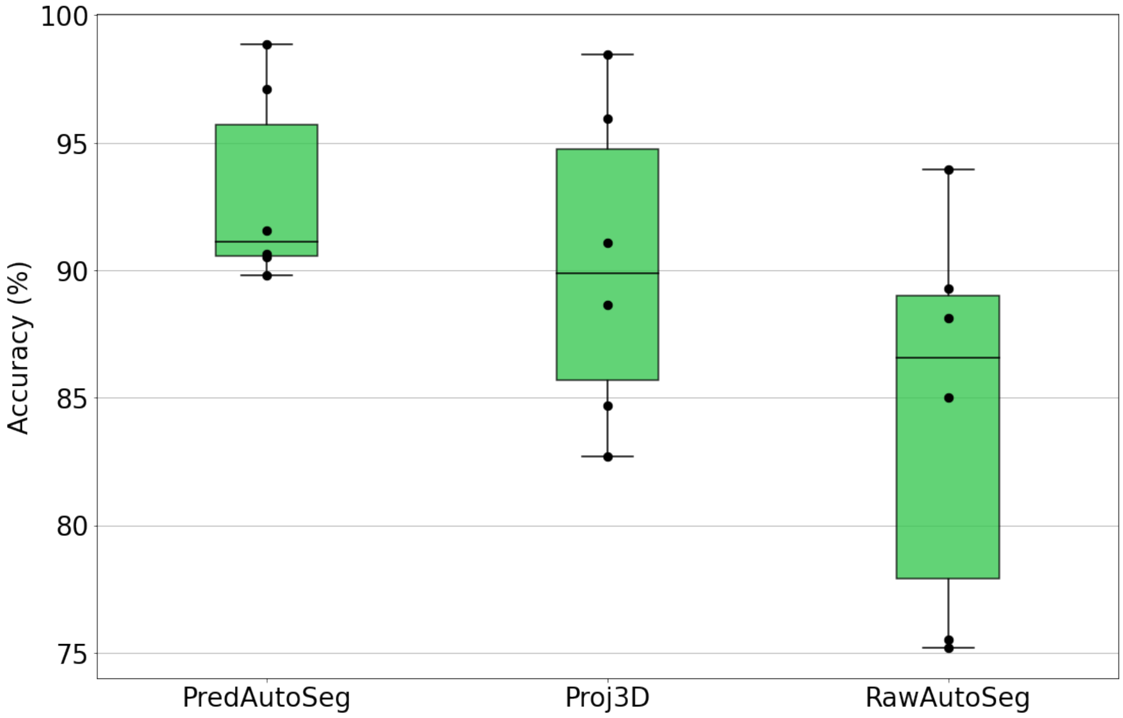
100
95
90
85
80
75
Accuracy (%)
PredAutoSeg
Proj3D
RawAutoSeg

[ ]:

```

```

[ ]:

```

```
