# Supplementary figures and images for: Accurate and versatile 3D segmentation of plant tissues at cellular resolution
Source: eLife. 2020 Jul 29;9:e57613. doi: 10.7554/eLife.57613 (PMC7447435; doi:10.7554/eLife.57613)

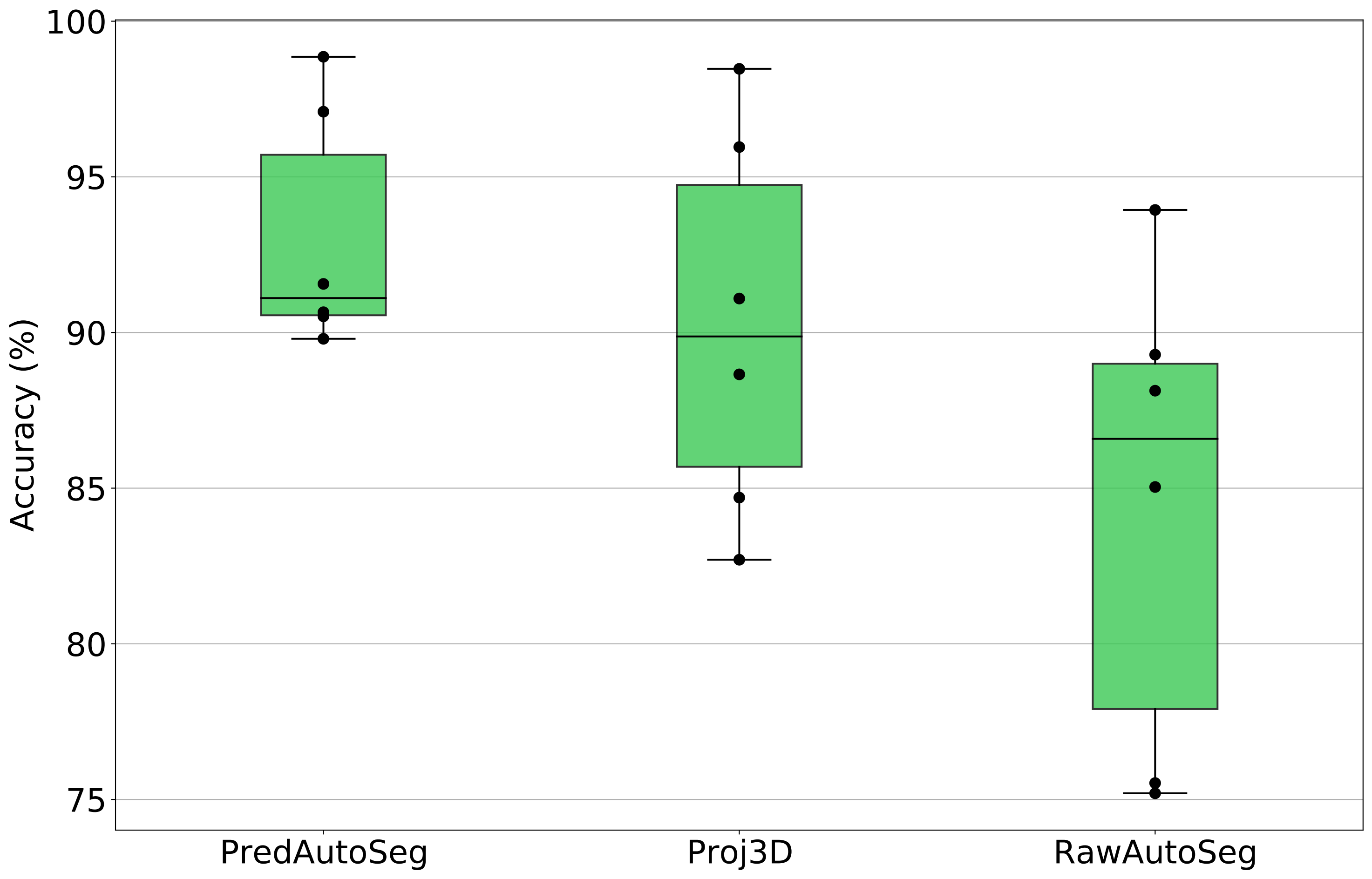

Supplement: Figure 9—source data 1. — The archive contains: 'final_mesh_evaluation - Sheet1.csv' - CSV file with evaluation scores computed on individual meshes, 'Mesh_boxplot.pdf' - detailed steps to reproduce the graphs, 'Mesh_boxplot.ipynb' - python script for generating the graph. [file elife-57613-fig9-data1.zip › figure9/figure_meshes/accuracy.pdf]

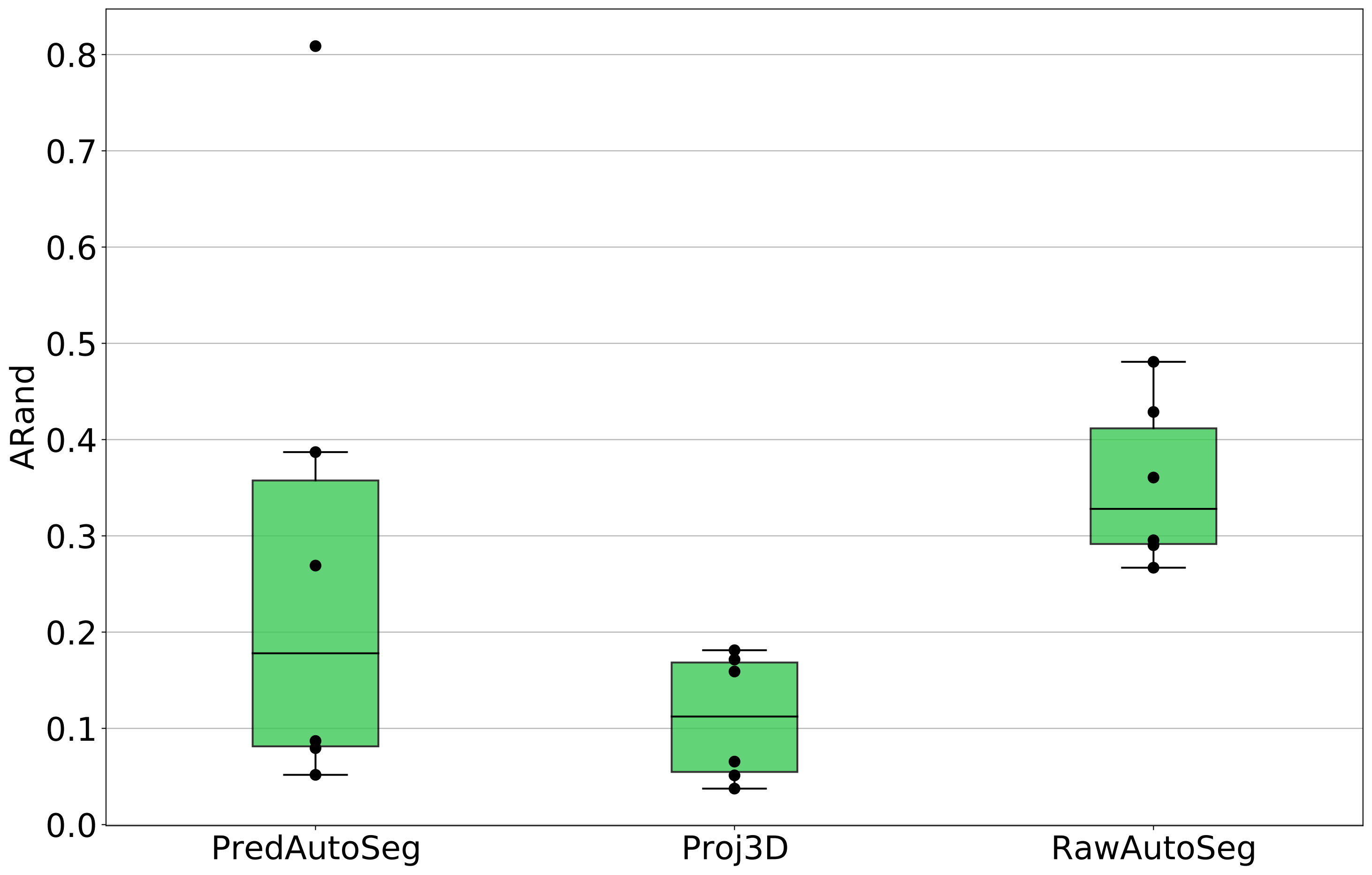

Supplement: Figure 9—source data 1. — The archive contains: 'final_mesh_evaluation - Sheet1.csv' - CSV file with evaluation scores computed on individual meshes, 'Mesh_boxplot.pdf' - detailed steps to reproduce the graphs, 'Mesh_boxplot.ipynb' - python script for generating the graph. [file elife-57613-fig9-data1.zip › figure9/figure_meshes/arand.pdf]
